# Supplementary material for: How seasons, weather, and part of day influence baseline affective valence in laboratory research participants?
Source: PLoS One. 2021 Aug 19;16(8):e0256430. doi: 10.1371/journal.pone.0256430 (PMC8376062; doi:10.1371/journal.pone.0256430)
Supplement: S1 Table — (DOCX) [file pone.0256430.s001.docx]

*Supplementary Materials*

| **Table S1**  *Overview of Studies Characteristics* | | | | | | | | | | | | | | |
| --- | --- | --- | --- | --- | --- | --- | --- | --- | --- | --- | --- | --- | --- | --- |
| Study ID | Sample characteristics | | | Study characteristics | | Frequencies | | | | | | | | Refe-rence |
|  | Size (% female) | Mean age (*SD*) | Participant pool | Study procedure | Duration | Winter | Spring | Autumn | Early morning | Late morning | Early afternoon | Late afternoon | Early evening |  |
| 1 | 192 (51) | 21.26 (2.09) | Under-graduates | Baseline (5 min), two rounds of sending gratitude or control message | 10.2016 – 02.2017 | 120 | 0 | 72 | 21 | 41 | 40 | 46 | 44 | [46] |
| 2 | 70 (49) | 21.07 (1.98) | Under-graduates | Baseline (5 min), six rounds of responding to the immoral success of other people (capitalization) | 11.2016 | 0 | 0 | 70 | 8 | 13 | 14 | 20 | 15 | [unpublished material] |
| 3 | 81 (0) | 21.34 (2.73) | Gamers | Baseline (5 min), two rounds of playing the video game | 03.2017 – 04.2017 | 6 | 75 | 0 | 6 | 12 | 19 | 17 | 27 | [47] |
| 4 | 222 (50) | 22.44 (2.63) | Romantic couples | Baseline (5 min), three rounds of watching emotional film clips, and responding to partner success (capitalization) | 01.2018 – 03.2018 | 222 | 0 | 0 | 12 | 22 | 62 | 64 | 62 | [48] |
| 5 | 177 (49) | 22.73 (2.99) | Romantic couples | Baseline (5 min), three rounds of completing the Navon task, receiving feedback from the romantic partner, and responding to partner success (capitalization) | 04.2018 – 05.2018 | 0 | 177 | 0 | 20 | 39 | 41 | 43 | 34 | [49] |
| 6 | 194 (53) | 21.58 (2.72) | Under-graduates | Baseline (5 min), watching six emotional film clips (12 min), playing the ultimatum game | 11.2018 – 12.2018 | 0 | 0 | 194 | 1 | 43 | 54 | 48 | 48 | [50] |
| 7 | 172 (49) | 21.81 (2.63) | Romantic couples | Baseline (5 min) four rounds of responding to partner immoral success (capitalization) | 02.2019 -03.2019 | 172 | 0 | 0 | 0 | 46 | 30 | 44 | 52 | [unpublished material] |
